# Targeted RNAi of BIRC5/Survivin Using Antibody-Conjugated Poly(Propylene Imine)-Based Polyplexes Inhibits Growth of PSCA-Positive Tumors

**DOI:** 10.3390/pharmaceutics13050676

**Published:** 2021-05-08

**Authors:** Willi Jugel, Achim Aigner, Susanne Michen, Alexander Hagstotz, Alexander Ewe, Dietmar Appelhans, Gabriele Schackert, Achim Temme, Stefanie Tietze

**Affiliations:** 1 Department of Neurosurgery, Section Experimental Neurosurgery and Tumor Immunology, University Hospital Carl Gustav Carus, TU Dresden, Fetscherstraße 74, 01307 Dresden, Germany; Willi.Jugel@uniklinikum-dresden.de (W.J.); Susanne.Michen@uniklinikum-dresden.de (S.M.); Alexander.Hagstotz@uniklinikum-dresden.de (A.H.); gabriele.schackert@uniklinikum-dresden.de (G.S.); achim.temme@uniklinikum-dresden.de (A.T.); 2 Rudolf-Boehm-Institute for Pharmacology and Toxicology, Clinical Pharmacology, Faculty of Medicine, University of Leipzig, 04107 Leipzig, Germany; achim.aigner@medizin.uni-leipzig.de (A.A.); alexander.ewe@medizin.uni-leipzig.de (A.E.); 3 Leibniz Institute of Polymer Research Dresden, Hohe Straße 6, 01069 Dresden, Germany; applhans@ipfdd.de; 4 German Cancer Consortium (DKTK), Partner Site Dresden, Fetscherstraße 74, 01307 Dresden, Germany; 5 German Cancer Research Center (DKFZ), In Neuenheimer Feld 280, 69120 Heidelberg, Germany; 6 National Center for Tumor Diseases (NCT), Fetscherstraße 74, 01307 Dresden, Germany

**Keywords:** targeted siRNA delivery, maltose-modified poly(propylene imine), Survivin, prostate stem cell antigen

## Abstract

Delivery of siRNAs for the treatment of tumors critically depends on the development of efficient nucleic acid carrier systems. The complexation of dendritic polymers (dendrimers) results in nanoparticles, called dendriplexes, that protect siRNA from degradation and mediate non-specific cellular uptake of siRNA. However, large siRNA doses are required for in vivo use due to accumulation of the nanoparticles in sinks such as the lung, liver, and spleen. This suggests the exploration of targeted nanoparticles for enhancing tumor cell specificity and achieving higher siRNA levels in tumors. In this work, we report on the targeted delivery of a therapeutic siRNA specific for BIRC5/Survivin in vitro and in vivo to tumor cells expressing the surface marker prostate stem cell antigen (PSCA). For this, polyplexes consisting of single-chain antibody fragments specific for PSCA conjugated to siRNA/maltose-modified poly(propylene imine) dendriplexes were used. These polyplexes were endocytosed by PSCA-positive 293T^PSCA/ffLuc^ and PC3^PSCA^ cells and caused knockdown of reporter gene firefly luciferase and Survivin expression, respectively. In a therapeutic study in PC3^PSCA^ xenograft-bearing mice, significant anti-tumor effects were observed upon systemic administration of the targeted polyplexes. This indicates superior anti-tumor efficacy when employing targeted delivery of Survivin-specific siRNA, based on the additive effects of siRNA-mediated Survivin knockdown in combination with scFv-mediated PSCA inhibition.

## 1. Introduction

Small interfering RNAs (siRNAs) can selectively target and downregulate tumor-relevant genes, holding great promise in cancer treatment. The siRNA-mediated silencing (RNAi) of genes utilizes a conserved biological process among multicellular organisms that mediates resistance to both endogenous parasitic and exogenous pathogenic nucleic acids, and regulates the expression of protein-coding genes. It relies on 21 bp to 23 bp double-stranded, so-called small interfering RNAs (siRNAs), whose guide strand is integrated into the RNA-induced silencing complex (RISC), which, upon sequence-specific binding, promotes the destruction of target mRNAs through an enzymatic activity integrated in the complex [1]. Importantly, the destruction of a specific cellular mRNA can also be achieved by transfection of chemically synthesized siRNA molecules, which then enter the RNAi pathway. The silencing by siRNA is highly efficient due to RISC-mediated protection of the guide strand from degradation and its catalytic activity, leading to the cleavage of many mRNA molecules [2].

SiRNA molecules are prone to degradation by serum nucleases, cannot easily cross membranes due to their size and negative net charge, and are subject to renal elimination. Therefore, in addition to chemical modification of siRNA [3], carrier systems have been established to increase siRNA half-life and transfection of cells [4]. Among non-viral systems for delivery of siRNA for cancer treatment (for review, see [5]), polymeric carrier molecules such as dendritic polymers poly(amidoamine) (PAMAM) and poly(propylene imine) (PPI) have emerged as suitable siRNA carriers [4,6]. Due to their cationic net charge, such polymeric carriers are capable of complexing negatively charged siRNA resulting in nanoparticles called dendriplexes. Tuning of polymeric carriers by surface modifications with sugars or poly(ethylene glycol) (PEG) is essential to reduce their cytotoxicity and to inhibit intermolecular aggregation of generated dendriplexes. Most importantly, such modifications provide a hydrophilic shell which reduces interaction with the reticuloendothelial system and therefore improve the circulation time in the bloodstream [7].

However, when considering PEG-modified carrier molecules for repeated delivery of siRNA, there is accumulating evidence of pre-existing as well as treatment-induced anti-PEG antibody responses. Several reports correlated the generation of anti-PEG antibodies with loss of therapeutic efficacy and described adverse effects after repeated administrations of PEGylated proteins, peptides and carrier molecules (for review, see [8]). Therefore, we have previously focused on sugar modifications, in particular maltose-modification of poly(ethylene imine) (PEI) and PPI carrier molecules as alternative for PEGylation [9]. Decreased positive net charges of dendriplexes upon more extensive sugar modification of the polymeric carriers, however, can also lead to a stealth-like mode, characterized by impaired cell membrane interaction and thus reduced siRNA delivery into cells [10]. On the other hand, this decrease in non-specific uptake efficacy provides the basis for targeted, ligand-mediated delivery and uptake. We recently described transfection-disabled maltose-modified PPI dendrimers (mPPI), which, upon coupling of antibodies for targeting, became competent for delivering siRNA to target cells expressing the cognate antigen/receptor. More specifically, we successfully developed a modular so-called polyplex system based on m-PPIs, neutravidin and bioconjugated mono-biotinylated single-chain antibodies (scFv) for targeted delivery of siRNA to EGFRvIII-positive tumors [10].

In the current study, we explore for the first time the targeted delivery of a therapeutic siRNA, namely Survivin-siRNA (siSurv), using an mPPI-based polyplex system with scFv-mediated targeting of PSCA, for treatment of PSCA-positive target cells and tumors.

Survivin belongs to the inhibitor of the apoptosis protein (IAP) family and is highly expressed in cancer tissues and cancer cell lines [11]. The anti-apoptotic activity of Survivin is achieved by reducing caspase activity. In particular, in cancer cells, Survivin sequesters the apoptosis-inducing factor Smac/DIABLO away from binding to the X-linked inhibitor of apoptosis (XIAP), which inhibits induction of intrinsic apoptosis by blocking Caspase 9 activation [12]. Furthermore, Survivin plays an additional and unique role in the regulation of mitotic events. It has been shown that chromosome segregation and cytokinesis essentially depend on Survivin [13,14]. Its functional relevance in the tumor and the fact that a disruption of the genetic Survivin locus cannot be compensated by other members of the IAP family [15] make Survivin a particularly attractive therapeutic RNAi target.

PSCA represents an associated glycophosphatidylinositol (GPI)-anchored cell surface antigen [16,17,18,19], which is expressed in normal prostate-specific tissue and overexpressed in prostate cancer specimens including both high-grade prostatic intraepithelial neoplasia and androgen-dependent/independent tumors [19]. PSCA is also expressed in prostate cancer metastases [20] and in prostate-unrelated carcinomas such as pancreatic adenocarcinoma [21], renal clear-cell carcinoma [22], transitional cell carcinoma [23], and glioblastoma [24]. Recently, we demonstrated successful delivery of TLR3 agonist Riboxxol-biotin conjugated to neutravidin/anti-PSCA exclusively to PSCA-positive target cells in vitro and in vivo [25]. We therefore considered PSCA as a relevant tumor-associated antigen which is a targetable structure for employing our polyplex approach for delivery of therapeutic siRNA.

In this study, we therefore investigate a novel polyplex system based on the combination of targeted delivery of therapeutic siRNA, siRNA-mediated Survivin knockdown and scFv-mediated PSCA inhibition. To specifically distinguish the extent to which each component contributes to the targeted siSurv delivery mediated by the PSCA-specific polyplexes, the in vivo anti-tumor effects were directly compared to those obtained with PSCA-specific polyplexes loaded with control siRNA (scFv-mediated PSCA inhibition only) and polyplexes loaded with siSurv but conjugated with a non-specific control antibody (Survivin knockdown only) [26]. Indeed, we found that the anti-tumor effects of the PSCA-targeted, siSurv-containing polyplexes significantly exceeded the anti-tumor effects of the single-component systems. Thus, the mPPI-based PSCA-targeted polyplexes provide an innovative platform for further exploration of targeted delivery of siRNAs and other nucleic acids for therapeutic application.

## 2. Materials and Methods

### 2.1. Synthesis of PPIs

Transfection-disabled maltose-modified PPI dendrimers (mPPI = mal19-PPI) and biotinylated mPPIs were synthesized and characterized as described previously [10]. Here, maltose-(mal)-modified fourth-generation PPI dendrimers (mal-PPI) were used with 19% (mal19-PPI) being modified (14,900 g/mol; modified with 24 maltose units per molecule).

### 2.2. Cell Lines

The human embryonic kidney cell lines 293T, 293T^huBirA^, 293T^huBirA-scFv(MR1.1)-P-BAP^ and 293T^PSCA^, the human HEK-Blue^hTLR3/PSCA^ SEAP reporter cell line as well as the prostate cancer cell lines PC3 and PC3^PSCA^ have been described previously [10,25,27]. The 293T^PSCA/ffLuc^ cell line with ectopic expression of the firefly luciferase was generated by lentiviral transduction of 293T^PSCA^ cells using pHATtrick-ffLuc-Puro vector. Packaging of viral particles and transduction were performed using a three-vector system described previously [28]. Transduced cells were selected with 20 μg/mL puromycin for 72 h. PC3 cell lines were maintained in RPMI-1640 completed with 10% *v/v* heat-inactivated FCS, 2 mM L glutamine, 100 µg/mL streptomycin, 100 U/mL penicillin, and 10 mM HEPES (all from Life Technologies). 293T and 293T^PSCA^ cell lines were cultured in DMEM completed with 4.5 g/L glucose, 10% *v/v* heat-inactivated FCS, 100 U/mL penicillin, 100 μg/mL streptomycin and 10 mM HEPES (all from Life Technologies). 293T^huBirA-scFv(MR1.1)-P-BAP^ and 293T^huBirA^ cells were maintained in DMEM complete supplemented with 50 μM Biotin-C6 (Sigma-Aldrich, Darmstadt, Germany) for scFv production. Human HEK-Blue^hTLR3/PSCA^ SEAP reporter cell line, expressing the human TLR3 gene was cultured in DMEM complete supplemented with 100 µg/mL normocin, 30 µg/mL blasticidin and 100 µg/mL zeocin (all antibiotics from Invivogen, San Diego, CA, USA). All cell lines were cultivated at 37 °C and 5% CO_2_ in a humidified incubator.

### 2.3. Vector Constructs and siRNA

The DNA sequence and features of single-chain antibody derivative scFv(AM1)-P-BAP and scFv (MR1.1)-P-BAP have been described previously [10]. Both constructs include an N-terminal Igκ leader sequence, a biotin acceptor peptide (P-BAP) and a C-terminal c-myc epitope and a 6x histidine (His)-tag. The siRNAs for knockdown of firefly luciferase (siLuc, 5′-CUUACGCUGAGUACUUCGA(tt)-3′, Eurofins MWG Biotech, Ebersbach, Germany) and human Survivin (siSurv, 5′-GAAUUAACCCUUGGUGAAU(tt)-3′, Eurofins MWG Biotech, Ebersbach, Germany) have been described previously [29].

### 2.4. Production of scFv-P-BAP and Polyplexes

The biotinylated scFv(AM1)-P-BAP was expressed in transiently transfected 293T^huBirA^ producer cells. The biotinylated scFv(MR1.1)-P-BAP was produced in 293T^huBirA-scFv(MR1.1)-P-BAP^ cells. The single-chain antibodies were purified from the harvested cell culture supernatants using Ni^2+^-NTA affinity chromatography. Briefly, 50 mL of clarified supernatant was passed through a Ni^2+^-NTA spin column (Qiagen, Hilden, Germany) and washed with 1X PBS containing 150 mM NaCl and 10 mM/20 mM imidazole. Elution of bound scFvs was performed in 500 μL 1X PBS containing 150 mM NaCl and 350 mM imidazole per column. Eluted scFvs were dialyzed in 1x PBS twice for 2 h and additionally for 12 h at 4 °C. The biotinylated scFv-P-BAPs were further purified using avidin-biotin affinity chromatography with monomeric avidin columns (Thermo Fisher Scientific, Waltham, MA, USA) according to the protocol of the manufacturer. Eluted scFvs were dialyzed again as described previously. The recombinant proteins were subsequently concentrated using Amicon tubes Ultra-15 (Merck Millipore, Burlington, MA, USA) and were stored in aliquots at −20 °C. Polyplexes were generated as described previously [10]. Briefly, polyplexes were built by sequential mixing of neutravidin, mono-biotinylated scFv-P-BAP and mono-biotinylated mal19-PPI-biotin using molar ratios 1:2:1. Saturation of remaining free biotin binding sites of neutravidin was accomplished with 0.3 mM D-biotin. Complexation between siRNA and mal19-PPI was achieved using a molar ratio 1:4. The electrostatic interactions between mal19-PPI-biotin/neutravidin/scFv-P-BAP and mal19-PPI/siRNA intermediate conjugates resulted in scFv(AM1)-P-BAP-guided polyplexes. After 24 h incubation at 4 °C, the generated polyplexes were used for the experiments.

### 2.5. Electrophoretic Mobility Gel Shift Assay

SiRNA (100 pmol) was incubated for 30 min at RT with increasing amounts of mal19-PPI corresponding to molar ratios of 1:1 to 1:80. For analysis of resistance to Rnase, siRNA/mal19-PPI-dendriplexes (1:5) were incubated with RNase A/T1 Mix (Thermo Fisher Scientific) for 30 min at 37 °C. The dendriplexes were then separated by agarose gel electrophoresis [3% (*w/v*)] and analyzed under UV light (G:Box Chemi XX9, VWR, Darmstadt, Germany).

### 2.6. Multiparameter Nanoparticle Tracking Analysis

Multiparameter nanoparticle tracking analysis was performed using the ZetaView® PMX120 (Particle Metrix GmbH, Inning am Ammersee, Germany) according to the manufacturer’s instructions to determine the zeta potential and the particle size of scFv(AM1)-P-BAP-polyplexes containing a control siRNA (siLuc). Polyplexes were prepared as described above. Data were analyzed using the manufacturer’s software (ZetaView 8.05.05).

### 2.7. RNA Interference and Western Blot Analysis

Produced scFv(AM1)-P-BAP and its conjugation to neutravidin was investigated using SDS-PAGE. For immunoblot analysis, 1 μg of scFv(AM1)-P-BAP was separated by SDS-PAGE (12% polyacrylamide gel) under reducing conditions. Proteins were transferred by semi-dry Western Blot to a PVDF membrane (Whatman). After blocking of the PVDF membrane with 5% non-fat dry milk in Tris-buffered saline containing 0.1% Tween 20 (TBS-T), the scFv-P-BAPs were detected using a primary monoclonal murine anti-c-myc antibody (1:5000, Invitrogen) and a secondary polyclonal rabbit anti-mouse IgG HRP conjugate (1:1000; Dako). Biotinylated scFv(AM1)-P-BAPs were detected by HRP-conjugated anti-biotin antibody (1:3000, Sigma-Aldrich). For determination of conjugated scFv(AM1)-P-BAP to neutravidin, the two components were mixed at various molar ratios of scFv-P-BAPs to neutravidin from 8:1 to 0.25:1 in 1x PBS for 30 min at room temperature. The polyplex-mediated knockdown of Survivin in PC3^PSCA^ cells was investigated using SDS-PAGE. Therefore, PC3^PSCA^ cells were consecutively transfected for three times every 8 h with scFv(AM1)-P-BAP polyplexes containing siSurv. Polyplexes containing a control siRNA (siLuc) were included as control. Interferin-mediated transfection of siSurv into PC3^PSCA^ cells was included to demonstrate the feasibility of siSurv for RNAi. Treated cells were lysed in lysis buffer (10 mM Tris-HCl, pH 8.0; 140 mM NaCl; 1% Triton X-100) 72 h after transfection. Cell lysates were cleared by centrifugation. Equal amounts of protein were subjected to SDS-PAGE under reducing conditions and blotted on PVDF membrane using semi-dry Western Blotting. After blocking PVDF membrane with 5% BSA in TBS-T, Survivin was detected using a polyclonal rabbit anti-human Survivin antibody (1:1000, R&D Systems, Minneapolis, MN, USA), followed by HRP-conjugated anti-rabbit IgG secondary antibody (1:1000; Dako). To demonstrate equal loading, PVDF membranes were subsequently stained using an anti-α tubulin antibody (1:5000; Sigma-Aldrich), followed by a secondary polyclonal rabbit anti-mouse IgG HRP conjugate (1:1000; Dako, Glostrup, Denmark). Visual capturing of proteins was performed by Luminata Forte Western HRP substrate (Merck Millipore) and G:Box Chemi XX9 (VWR) gel doc system and analyzed by Fiji software (ImageJ 1.51k, National Institute of Health).

#### Clonogenic Survival Assay

To determine frequency of clonogenic survival, 1 × 10^3^ PC3^PSCA^ cells incubated with scFv(AM1)-P-BAP/neutravidin conjugates for 24 h were plated in 10 cm plates. Cells incubated with scFv(MR1.1)-P-BAP/neutravidin conjugates were included as negative control. At day 14, cells were stained with Giemsa staining solution (Merck KGaA, Darmstadt, Germany) and adherent colonies were counted.

### 2.8. Flow Cytometry

Binding of biotinylated scFv(AM1)-P-BAP to PSCA-positive target cells was analyzed by flow cytometry analysis after staining of 2 × 10^5^ cells with 5 μg scFv-P-BAP for 1 h at 4 °C, followed by secondary anti-biotin-VioBlue antibody (Miltenyi Biotec, Bergisch Gladbach, Germany). Cells stained only with secondary antibody were included as control. To assess siRNA uptake driven by scFv-P-BAP-polyplexes, 2 × 10^5^ 293T^PSCA/ffLuc^ cells were incubated with Cy3-labelled polyplexes for 4 h at 37 °C. Subsequently, cells were washed with 0.1% Heparin/PBS (Sigma-Aldrich). Cy3-labelled polyplexes with scFv(MR1.1)-P-BAP and without scFv were included as negative controls. Interferin (Polyplus transfection)-mediated uptake of Cy3-labelled siRNA was comprised as positive control. To determine cell proliferation, 1 × 10^5^ PC3^PSCA^ cells labelled with carboxyfluorescein succinimidyl ester (CFSE, BioLegend) were incubated with scFv(AM1)-P-BAP/neutravidin conjugates or scFv(MR1.1)-P-BAP/neutravidin conjugates using molar ratios of 2:1. The cells were cultivated 1–7 days prior to analysis by flow cytometry, and crosslinking with the scFv-P-BAP/neutravidin conjugates was repeated every 24 h. At least 2 × 10^4^ cells were measured by MACSQuant Analyzer 10 flow cytometer (Miltenyi Biotec) and analyzed by FlowJo software version 10.1 (TreeStar Inc., Ashland, OR, USA).

### 2.9. Confocal Laser Scanning Microscopy

For visualization of cellular siRNA uptake, 6 × 10^5^ 293T^PSCA/ffLuc^ cells grown on a coverslip were incubated with scFv(AM1)-P-BAP-polyplexes loaded with Cy3-labelled siRNA for 24 h at 37 °C. After fixation of cells with 4% paraformaldehyde in PBS, cell membranes and nuclei were stained with Alexa Fluor 647 conjugated Wheat Germ Agglutinin (WGA, Life Technologies, Carlsbad, CA, USA) and Hoechst 33,342 (Invitrogen, Carlsbad, CA, USA) according to the manufacturers protocols. The coverslips were placed upside down in a drop of mounting medium (Vector Laboratories) on a microscope slide. Cy3-labelled polyplexes conjugated with control scFv(MR1.1)-P-BAP, which cannot bind to 293T^PSCA/ffLuc^ cells or without conjugation of scFv-P-BAP were included as negative controls. The images were captured by a confocal laser scanning microscope (Leica SP5, Leica, Wetzlar, Germany) and analyzed by Fiji software (ImageJ 1.51k, National Institute of Health).

### 2.10. Determination of Luciferase Activity

For testing specific luciferase knockdown when using PSCA-specific polyplexes delivering siRNA for luciferase (siLuc), 1.5 × 10^5^ 293T^PSCA/ffLuc^ cells were transfected with scFv(AM1)-P-BAP polyplexes containing siLuc. For assessing the route of internalization, 0.6 μg/mL filipin III and 6.0 μg/mL chlorpromazine (both Sigma-Aldrich) were added 1 h prior polyplex treatment. In all experiments, polyplexes loaded with siLuc, but conjugated to a control scFv-P-BAP (scFv(MR1.1)-P-BAP, specific for EGFRvIII) and polyplexes loaded with control siRNA specific for red fluorescent protein (siRFP1) conjugated to scFv(AM1)-P-BAP or scFv(MR1.1)-P-BAP served as negative controls. Interferin-mediated transfection of siLuc was included as positive control for RNAi of luciferase. Luciferase activities of total cell lysates were measured 72 h after transfection using the Luciferase Assay System (Promega) according to the protocol of the manufacturer. Chemiluminescence was measured using Synergy 2 Multi-Mode Microplate Reader (BioTek Instruments, Winooski, VT, USA). The luciferase knockdown efficiency of the different polyplexes was normalized to the corresponding siRFP1-treated controls using the formula: knockdown efficiency [%] = 100 − ((relative light units (RLU)siLuc/RLUsiRFP1) × 100).

### 2.11. Analysis of Human TLR3 Activation

To monitor human TLR3 activation by PSCA-specific polyplexes loaded with Survivin siRNA, the HEK-Blue detection system was used as described previously [25]. Briefly, 5 × 10^4^ HEK-Blue^hTLR3/PSCA^ cells were incubated with PSCA-specific polyplexes based on 25 pmol siRNA. The TLR3 ligand poly(I:C) (1 µg/mL, Sigma-Aldrich) was included as positive control. After 24 h, hydrolysis of secreted alkaline phosphatase (SEAP) color substrate (InvivoGen) by SEAP was quantified at 655 nm using Synergy 2 Multi-Mode Microplate Reader (BioTek Instruments).

### 2.12. Delivery of PSCA-Specific Polyplexes to Mice Bearing Human Prostate Cancer Xenografts

The animal experiments were approved by the Landesdirektion Sachsen (animal permission number: TVV 21/13). The methods were carried out in accordance with the approved guidelines and regulations. 2 × 10^6^ PC3^PSCA^ cells were subcutaneously implanted into the right and left flank of athymic nude mice (NMRI^Foxn1nu/Foxn1nu^). After reaching an average tumor diameter of 2.5 mm, mice were injected intraperitoneally with PSCA-specific polyplexes containing 10 µg siSurv, 80.8 µg mal19-PPI, 15.6 µg mal19-bPPI, 43.8 µg neutravidin, 35.9 µg scFv(AM1)-P-BAP and 1.8 µg D-biotin every three days for 17 days. Mice injected with PSCA-specific polyplexes loaded with negative control siRNA specific for luciferase (siLuc), with EGFRvIII-specific polyplexes containing siSurv or with EGFRvIII-specific polyplexes containing siLuc were included as negative controls. Tumor sizes were determined by measuring all three dimensions.

### 2.13. Statistical Analysis

All experiments were performed at least three times. Differences between groups were examined for statistical significance using Student’s *t*-test. Values of *p* < 0.05 were considered as statistically significant: * *p* < 0.05, ** *p* < 0.01, and *** *p* < 0.001.

## 3. Results

### 3.1. Synthesis and Characterization of scFv(AM1)-P-BAP Molecules

**Figure 1:**
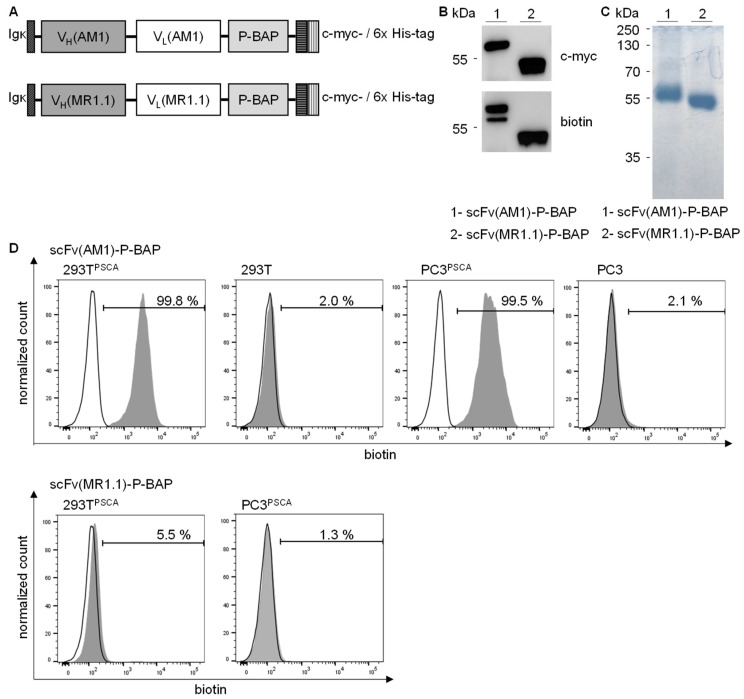
Production and characterization of recombinant biotinylated scFv(AM1)-P-BAP and scFv(MR1.1)-P-BAP. (**A**) Schematic presentation of the scFv(AM1)-P-BAP and control antibody scFv(MR1.1)-P-BAP protein domains. (**B**) Western Blot analysis of biotinylated scFv(AM1)-P-BAP and scFv(MR1.1)-P-BAP using anti-c-myc and anti-biotin antibodies. (**C**) Coomassie Brilliant Blue-stained polyacrylamide gel of purified scFv(AM1)-P-BAP and scFv(MR1.1)-P-BAP recombinant antibody derivatives. (**D**) Flow cytometry analysis of 293T^PSCA^, 293T wild-type, PC3^PSCA^ and PC3 wild-type cells stained with scFv(AM1)-P-BAP or scFv(MR1.1)-P-BAP. Binding of the scFvs was detected by secondary anti-biotin-VioBlue (grey histograms). Open histograms represent control staining using only secondary antibody.

Here, we explored a biotin-neutravidin-based, so-called polyplex modular system for anti-PSCA scFv antibody-mediated delivery of poly(propylene imine) carrier molecules containing Survivin-specific siRNA to PSCA-positive tumor cells. For this, we established a PSCA-specific scFv(AM1), fused to a biotin acceptor peptide sequence (P-BAP), and an similarly structured EGFRvIII-specific scFv(MR1.1)-P-BAP as negative control [10]. The structures of the scFv(AM1)-P-BAP and scFv(MR1.1)-P-BAP are shown in Figure 1A. All antibody constructs contained an N-terminal Igκ chain leader sequence for the extracellular secretion as well as a C-terminal c-myc-epitope and a 6x histidine (His)-tag for detection and purification, respectively. The biotinylated scFv-P-BAPs were purified from clarified cell culture supernatant of antibody-expressing 293T^huBirA^ cells using Ni^2+^-NTA affinity chromatography, followed by biotin affinity chromatography to obtain only biotinylated scFv-P-BAPs. In addition to the detection of the c-myc-epitope in Western Blot analysis, a biotin-specific antibody demonstrated the successful biotinylation of scFv(AM1)-P-BAP and the control antibody scFv(MR1.1)-P-BAP (Figure 1B). The observed bands match the calculated molecular mass of roughly 55 kDa. The Coomassie Brilliant Blue stained polyacrylamide gel shown in Figure 1C confirmed the purity and successful expression of the full-length proteins (Figure 1C). The binding properties of the scFv(AM1)-P-BAP were characterized by flow cytometry (Figure 1D). Results clearly demonstrated highly efficient binding of the scFv(AM1)-P-BAP to 293T^PSCA^ and PC3^PCSA^ cells, while as expected, the PSCA-specific single-chain antibody failed to bind to 293T and PC3 wild-type cells. Since a biotin-specific antibody was used as a secondary antibody, we were able to show that the C-terminal biotin residue was accessible under native conditions. As expected, the EGFRvIII-specific control antibody did not bind PSCA-positive 293T cells or PSCA-positive PC3 cells (Figure 1D).

### 3.2. Assembly of scFv(AM1)-P-BAP-Guided Polyplexes

**Figure 2:**
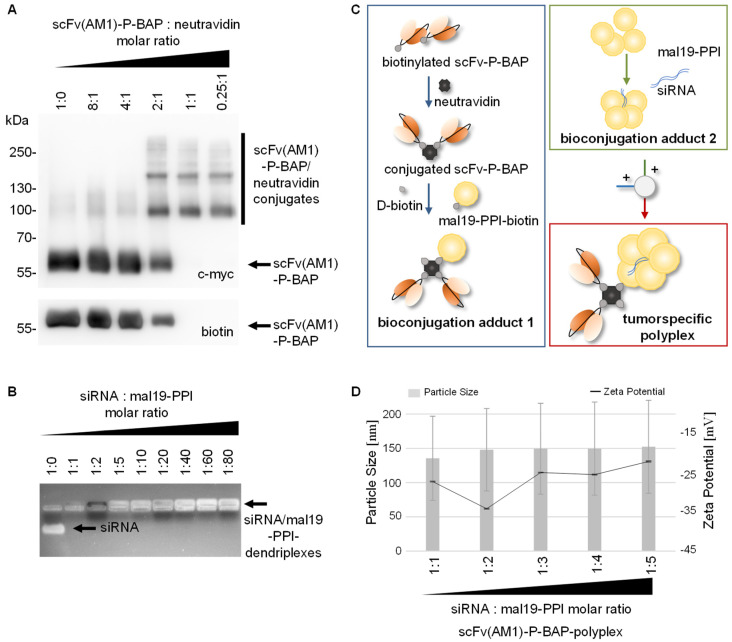
Assembly of scFv(AM1)-P-BAP-guided polyplexes. (**A**) Titration for the binding of scFv(AM1)-P-BAP to neutravidin with increasing molar ratios. Western Blot analysis showing scFv(AM1)-P-BAP/neutravidin complex formation at approximately 100 and 160 kDa or unbound scFv(AM1)-P-BAP at approximately 55 kDa using anti- c-myc and anti-biotin antibodies. (**B**) Electrophoretic mobility gel shift assay of siRNA binding to mal19-PPI. (**C**) Schematic representation of the successive conjugation of scFv(AM1)-P-BAP-guided polyplexes (**D**) Assessment of particle sizes and zeta potential of scFv(AM1)-P-BAP-polyplexes at various siRNA/mal19-PPI molar ratios.

The stable conjugation of scFv(AM1)-P-BAP to neutravidin was proven by Western Blot analysis shown in Figure 2A. A constant amount of scFv(AM1)-P-BAP was incubated with neutravidin at molar ratios 8:1, 4:1, 2:1, 1:1 and 0.25:1. Due to the high affinity of neutravidin to biotin, the conjugates remained stable during SDS-PAGE. The scFv(AM1)-P-BAP/neutravidin conjugates were detected by their C-terminal c-myc-epitope of the scFv-P-BAP. Distinct scFv(AM1)-P-BAP/neutravidin conjugate bands were detected at approximately 100 and 160 kDa for molar scFv/neutravidin ratios 2:1, 1:1 and 0.25:1. Non-conjugated scFv(AM1)-P-BAP containing an accessible biotin residue were detected by an anti-biotin antibody at approximately 55 kDa for molar scFv/neutravidin ratios 8:1, 4:1 and 2:1. In contrast, free biotinylated scFv(AM1)-P-BAP was absent when using scFv/neutravidin molar ratios of 1:1 and 0.25:1. The proper complexation of siRNA with mal19-PPI was confirmed by electrophoretic gel retardation assay shown in Figure 2B. Of note, all siRNA:mal19-PPI molar ratios ranging from 1:1 to 1:80 led to full complexation of siRNA with the polycationic mal19-PPI, as indicated by the absence of free siRNA migrating in the electrical field. Furthermore, RNase protection assay confirmed resistance of mal19-PPI/siRNA-dendriplexes to RNAse A/T1 (Supplementary Figure S1). To generate tumor-specific polyplexes delivering siRNA to target cells with cognate receptor expression, neutravidin was conjugated to mono-biotinylated scFv-P-BAP and mono-biotinylated mal19-PPI-biotin using molar ratios 1:2:1, respectively. To avoid potential biotin-driven agglutination through unwanted crosslinking of the components, the remaining biotin-binding pockets of neutravidin were saturated after conjugation of scFv-P-BAPs and mal19-PPI-biotin by adding D-biotin. Complexation between siRNA and mal19-PPI was achieved using a molar ratio 1:4. The electrostatic interactions between mal19-PPI-biotin/neutravidin/scFv-P-BAP (bioconjugation adduct (1) and mal19-PPI/siRNA (bioconjugation adduct (2) intermediate conjugates resulted in scFv(AM1)-P-BAP-guided polyplexes (Figure 2C). Assessment of the physiochemical properties of scFv(AM1)-P-BAP-polyplexes revealed a negative surface charge (−26.7 mV) and average particle size of 135.5 nm for molar siRNA/mal19-PPI ratio 1:1. With increasing mal19-PPI macromolecules incorporated in scFv(AM1)-P-BAP-polyplexes zeta potential and particle size increased towards −21.5 mV and 152.3 nm, respectively, for a molar siRNA/mal19-PPI ratio of 1:5.

### 3.3. Targeted Delivery of siRNA in PSCA-Positive Tumor Cells

**Figure 3:**
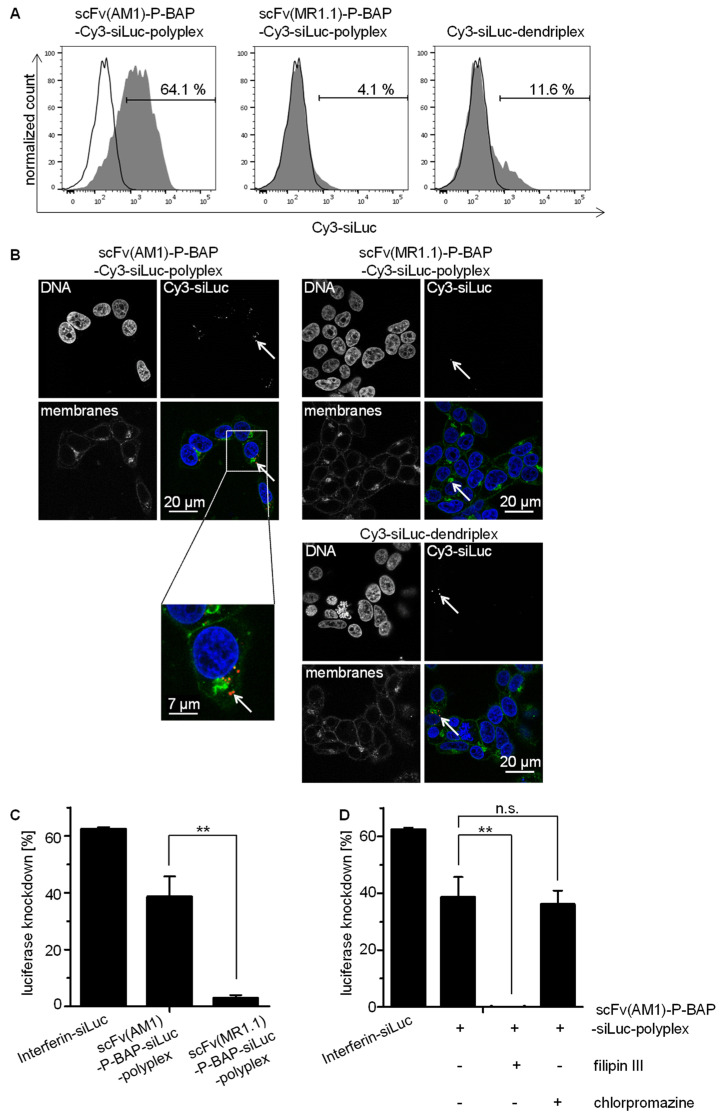
Targeted delivery of scFv(AM1)-P-BAP-guided polyplexes in PSCA-positive cells. (**A**) 293T^PSCA/ffLuc^ cells were treated with scFv(AM1)-P-BAP or scFv(MR1.1)-P-BAP polyplexes and scFv-P-BAP-free dendriplexes containing Cy3-labelled siRNA for 4 h (grey histograms). As control, untreated 293T^PSCA/ffLuc^ cells were utilized (open histograms). After Heparin-washing of surface-bound antibodies, the internalized Cy3-labelled siRNA was measured by flow cytometry. (**B**) Confocal laser scanning microscopy analysis of 293T^PSCA/ffLuc^ cells treated with scFv(AM1)-P-BAP or scFv(MR1.1)-P-BAP polyplexes and scFv-P-BAP-free dendriplexes containing Cy3-labelled siRNA. Arrows depict Cy3-labelled siRNA. (**C**) Knockdown efficiencies of luciferase activities in 293T^PSCA/ffLuc^ cells treated with scFv(AM1)-P-BAP-guided and scFv(MR1.1)-P-BAP-guided polyplexes containing siLuc. As positive RNAi control, cells were transfected with siLuc using the transfection reagent interferin (*n* = 2, mean ± SD, ** *p* ˂ 0.01). (**D**) Knockdown efficiencies of luciferase activity in 293T^PSCA/ffLuc^ cells treated with scFv(AM1)-P-BAP-guided and scFv(MR1.1)-P-BAP-guided polyplexes containing siLuc in the presence of inhibitors of endocytosis filipin III and chlorpromazine. As positive RNAi control, cells were transfected with siLuc using the transfection reagent interferin (*n* = 3, mean ± SD, ** *p* ˂ 0.01).

For characterization of targeted siRNA delivery to tumor cells, 293T^PSCA/ffLuc^ were treated with scFv(AM1)-P-BAP-guided polyplexes. As negative controls, non-specific polyplexes containing the EGFRvIII-specific scFv(MR1.1)-P-BAP or dendriplexes without scFv-P-BAP were included. All poly- and dendriplexes contained a Cy3-labelled siRNA (Cy3-siLuc) in order to allow detection of siRNA delivery into cells. Flow cytometry analysis, shown in Figure 3A, demonstrated a scFv(AM1)-P-BAP-mediated internalization of the polyplexes by 293T^PSCA/ffLuc^ cells, whereas no Cy3 signals were detectable after treatment with non-specific dendriplexes devoid of scFv-P-BAP or polyplexes conjugated with scFv(MR1.1)-P-BAP, respectively. This indicates that the siRNA delivery is enabled by receptor-mediated endocytosis. The right histogram of Figure 3A shows a moderate unspecific internalization of dendriplexes (devoid of scFv-P-BAP) which is most likely mediated by electrostatic interaction between polyplexes and the cell membrane. Confocal laser scanning microscopy studies, shown in Figure 3B, supported the data obtained from flow cytometry analysis. The tumor-specific scFv(AM1)-P-BAP-Cy3-siLuc-polyplexes were found to be internalized by 293T^PSCA/ffLuc^ cells. Well recognizable, the Cy3-labelled siRNA was located intracellularly in the cytoplasm, but not on the outer membrane, thus confirming cell internalization. In contrast, the scFv(MR1.1)-P-BAP-guided polyplexes as well as the scFv-P-BAP-free dendriplexes show essentially no nanoparticle uptake. The endosomal release and knockdown potential of polyplex-mediated luciferase-specific siRNA in 293T^PSCA/ffLuc^ cells was determined by a luciferase assay (Figure 3C). Of note, PSCA-positive target cells treated with scFv(AM1)-P-BAP-guided siLuc-containing polyplexes revealed a significant 39% decrease in luciferase activity, while luciferase knockdown of control polyplexes conjugated to non-binding scFv(MR1.1)-BAP was less than 3%. The low knockdown activities of the non-specific control polyplexes thus coincided with the data from flow cytometry and confocal laser scanning microscopy analysis shown above. To analyze the receptor mediated uptake of scFv(AM1)-P-BAP-guided polyplexes in more detail, the same experiments were conducted in the presence of inhibitors of endocytosis. In general, endocytosis of human cells can be differentiated between clathrin-dependent and clathrin-independent endocytosis. Clathrin-dependent endocytosis, which can be blocked by chlorpromazine [30], is characterized by the formation of clathrin-coated vesicles from clathrin-coated pits, which then enter the endosomal pathway [31]. In contrast, clathrin-independent endocytosis involves caveolae-dependent and lipid raft-mediated mechanisms that are inhibited by filipin III [32]. Clearly, the filipin III treatment of 293T^PSCA/ffLuc^ cells during incubation with scFv(AM1)-P-BAP-guided polyplexes abolished RNAi-mediated luciferase knockdown, while endocytosis and gene knockdown were not inhibited by treatment with chlorpromazine. This identifies clathrin-independent endocytosis as the uptake mechanism.

### 3.4. PSCA-Specific Polyplexes Delivering Survivin siRNA Exert Anti-Tumor Effects in PC3^PSCA^ Xenograft-Bearing Mice

**Figure 4:**
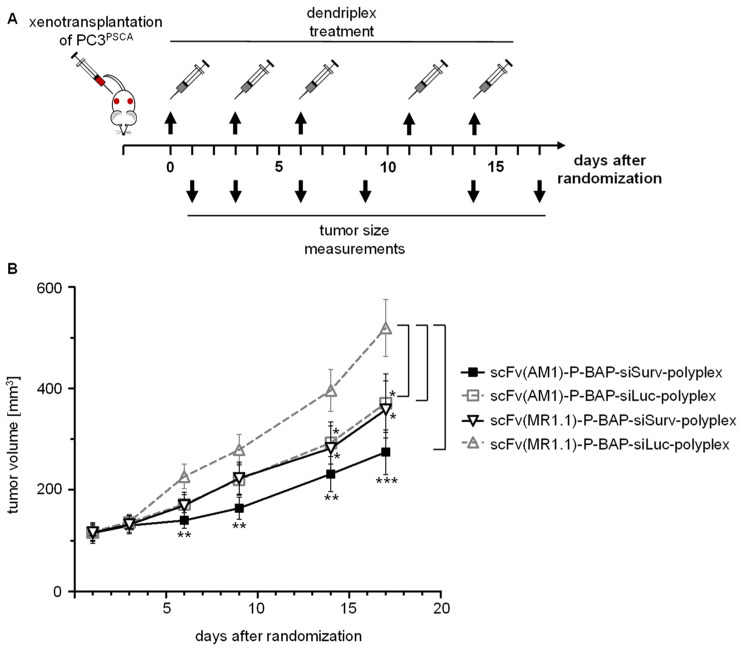
Targeted delivery of therapeutic siRNA using scFv(AM1)-P-BAP-guided polyplexes in PSCA-positive PC3 xenografts. (**A**) To analyze the targeted delivery of therapeutic siRNA, scFv(AM1)-P-BAP-guided polyplexes containing siSurv were injected intraperitoneally (i.p.) every third day for 17 days into PC3^PSCA^ tumor-bearing mice. As control, mice were treated with scFv(AM1)-P-BAP-guided polyplexes containing siLuc and control scFv(MR1.1)-P-BAP-guided polyplexes containing siSurv or siLuc. Tumor volume was evaluated one day after the injection of polyplexes. (**B**) Curve graph showing tumor volume in PC3^PSCA^ tumor-bearing mice after i.p. injection of polyplexes over time (*n* = 5, mean ± SEM, * *p* < 0.05, ** *p* < 0.01, and *** *p* < 0.001).

For the exploration of our system in a more relevant therapeutic in vivo situation, we investigated the efficacy of targeted RNAi-mediated Survivin knockdown in PC3^PSCA^ xenografts in NMRI^Fox1nu/Fox1nu^ mice. The efficient knockdown of Survivin expression upon successful delivery of siSurv into PC3^PSCA^ cells by PSCA-specific polyplexes was first proven in vitro, with Survivin knockdown almost reaching efficiencies of interferin as classical reagent for in vitro siRNA transfection (Supplementary Figure S2). This also demonstrates the efficacy of the selected siRNA. For in vivo therapy, the scFv(AM1)-P-BAP-guided polyplexes containing the Survivin-specific siRNA (siSurv) were injected intraperitoneally (i.p.) every third day for 17 days into PC3^PSCA^ tumor-bearing mice (Figure 4A). Remarkably, this treatment with siSurv-loaded scFv(AM1)-P-BAP-guided polyplexes led to a profound, ~50% inhibition of subcutaneous tumor growth when compared to the double-negative control group, i.e., mice treated in the same way with polyplexes containing the non-binding scFv(MR1.1)-P-BAP and negative control siLuc (Figure 4B). Notably, however, anti-tumor effects were also observed in mice treated with polyplexes containing the PSCA targeting antibody scFv(AM1)-P-BAP plus negative control siRNA (siLuc) or treated with non-targeted, scFv(MR1.1)-P-BAP-guided polyplexes loaded with siSurv. While these therapeutic effects reached statistical significance as well, they were less profound compared to the double-specific treatment group. To exclude anti-tumor activity of polyplexes due to siRNA-mediated activation of Toll-like receptor 3 (TLR3) [33,34], PSCA-engineered HEK-Blue^hTLR3/PSCA^ reporter cells were treated with polyplexes loaded with siSurv. As shown in Supplementary Figure S3, the potent TLR3 ligand poly(I:C) induced SEAP expression in the reporter cells, whereas internalized scFv(AM1)-P-BAP polyplexes and the non-internalized scFv(MR.1.1)-P-BAP-polyplexes, both loaded with siSurv, did not induce an innate cellular immune response. Thus, it can be concluded that the siRNAs and their complexation with mPPI did not interfere with TLR3 signaling and that the moderate anti-tumor effects observed after systemic administration of polyplexes containing the control antibody in PC3^PSCA^ tumor-bearing mice are based on the non-targeted delivery of siSurv. Likewise, the results of this study demonstrate moderate anti-tumor activity of PSCA antibodies, which is in line with previous reports [18] and contributes to the anti-tumor effects of our system.

## 4. Discussion

Since the discovery of RNAi as a powerful method for gene silencing [35], RNAi effector molecules (e.g., synthetic siRNA) have been applied in numerous clinical trials [36], which has more recently led to the approval of the first siRNA therapeutics (Patisiran, Givosiran). However, before RNAi-based therapeutic approaches can be applied, significant hurdles still need to be overcome, such as the development of efficient delivery systems. Recently, we established a novel tumor-specific siRNA delivery system, consisting of maltose-modified-PPI/PPI-biotin, neutravidin and a biotinylated scFv for targeted siRNA delivery to tumor cells [10]. Here, we investigated this modular platform technology with regard to the combination of a therapeutic siRNA for knockdown of a relevant target (Survivin) with a PSCA-specific scFv that simultaneously functions as a targeting moiety and PSCA inhibitor. PSCA was chosen since it is found on a broad range of tumor entities [20,21,22,23]. Recent studies from our group have established PSCA as a targetable antigen, which after receptor crosslinking by anti-PSCA immunoconjugates induces endocytosis [25]. In general, two main pathways for receptor-mediated endocytosis can be distinguished: clathrin-dependent and caveolae-dependent and lipid raft-mediated endocytosis. In this study, we identify caveolae/lipid raft-mediated endocytosis, but not clathrin-mediated endocytosis as the mechanism of PSCA-specific polyplex internalization. These findings are in line with previous studies, that demonstrated involvement of caveolae-dependent endocytosis in the internalization of other GPI-anchored proteins [16,17,18,19,37]. Yet, these results are in contrast to our recent report which revealed a mixed clathrin- and caveolae-mediated uptake of nanoparticle-like immunoconjugates comprising mono-biotinylated anti-PSCA-scFv conjugated via neutravidin to mono-biotinylated TLR3 agonist [25]. This discrepancy may be due to differences in nanoparticle sizes, since the nanoparticle-like immunoconjugates were estimated to be in the range of 22–55 nm in size, whereas the particle sizes of polyplexes were determined at 84–220 nm [23]. It remains to be investigated how the size of nanoparticles will indeed affect the mode of receptor-mediated internalization.

It is unanimously recognized that endosomal release of siRNA into cytoplasm is one of the key hurdles for most siRNA and nucleic acid delivery systems, often limiting their therapeutic effects. Notably, we demonstrated RNAi-mediated luciferase knockdown in 293T^PSCA/ffLuc^ reporter cells after targeted delivery of siLuc using PSCA-specific polyplexes, indicating efficient endosomal escape of siRNA molecules. Concomitantly, PSCA-specific polyplexes delivering siSurv showed profound downregulation of Survivin in PC3^PSCA^ prostate cancer cells in vitro. In line with this, our in vivo administration of PSCA-specific polyplexes in human PC3^PSCA^ xenograft-bearing mice led to a significant inhibition of tumor growth. Of note, PSCA-targeted polyplexes containing siLuc control siRNA resulted in moderate anti-tumor effects as well. This is in line with previous studies reporting that anti-PSCA monoclonal antibodies derived from hybridoma 1G8 significantly inhibited the growth of established orthotopic tumors and prolonged the survival of mice bearing human prostate cancer xenografts [18,38]. Although our scFv was derived from a different hybridoma (7F5) [39], it is conceivable that the scFv(AM-1)-P-BAP antibody derivative when embedded in a polyplex might induce some inhibitory effects on PSCA-expressing cells. Yet, treatment of PC3^PSCA^ cells with PSCA-specific polyplexes loaded with siLuc had no effects on apoptosis or clonal survival of PC3^PSCA^ cells in vitro (Supplementary Figures S4 and S5). This also indicates that classical 2D cell culture systems may only insufficiently monitor tumor-inhibitory effects of anti-PSCA antibodies, which can also rely on the modulation of other processes critical for tumor growth like tumor cell differentiation [40], modulation of angiogenesis factors [41] or disruption of cell–cell or cell-matrix interaction [42]. Moderate anti-tumor effects were also observed after systemic administration of control polyplexes loaded with Survivin-siRNA. This may well be due to the non-targeted delivery of the siRNA, probably facilitated by the enhanced permeability and retention (EPR) effect [43], that is often observed in tumors and can support the accumulation of nanoparticles in tumor tissue.

## 5. Conclusions

In summary, we successfully validated a modular platform technology for the targeted delivery of therapeutic siRNA to tumor cells. In succeeding experiments, it is mandatory to investigate potential beneficial effects but also limitations of tumor targeting polyplexes to pave the way for translation into the clinics. In particular, studies using syngeneic tumors models are envisioned to investigate potential immune responses due to facilitated release of tumor antigens after treatment with polyplexes delivering therapeutic siRNAs.

## Data Availability

The data presented in this study are available on request from the corresponding author.

## Supplementary Materials

The following are available online at https://www.mdpi.com/article/10.3390/pharmaceutics13050676/s1, Figure S1: RNase protection assay on the resistance of siRNA/mal19-PPI-dendriplex (1:5) on RNase A/T1 Mix. Figure S2: Targeted delivery of therapeutic siRNA using scFv(AM1)-P-BAP-guided polyplexes in PSCA-positive PC3 cells. Figure S3: HEK-Blue^hTLR3/PSCA^ cells were treated with scFv(AM1)-P-BAP-guided polyplexes containing siSurv. Figure S4: Clonogenic survival assay of PC3^PSCA^ cells after treatment with scFv(AM1)-P-BAP/neutravidin conjugates compared to scFv(MR1.1)-P-BAP/neutravidin conjugates (*n* = 3, mean ± SD). Figure S5: Carboxyfluorescein succinimidyl ester (CFSE)-based cell proliferation assay of PC3^PSCA^ cells after treatment with scFv(AM1)-P-BAP/neutravidin or scFv(MR1.1)-P-BAP-conjugates.
