# Supplementary material for: Targeted RNAi of BIRC5/Survivin Using Antibody-Conjugated Poly(Propylene Imine)-Based Polyplexes Inhibits Growth of PSCA-Positive Tumors
Source: Pharmaceutics. 2021 May 8;13(5):676. doi: 10.3390/pharmaceutics13050676 (PMC8151203; doi:10.3390/pharmaceutics13050676)
Supplement: Supplementary file 1 [file pharmaceutics-13-00676-s001.zip › pharmaceutics-1203062-supplementary.pdf]

pharmaceutics

Supplementary Materials: Targeted RNAi of BIRC5/Survivin Using Antibody-Conjugated Poly(Propylene Imine)-Based Polyplexes Inhibits Growth of PSCA-Positive Tumors

Willi Jugel, Achim Aigner, Susanne Michen, Alexander Hagstotz, Alexander Ewe, Dietmar Appelhans, Gabriele Schackert, Achim Temme and Stefanie Tietze

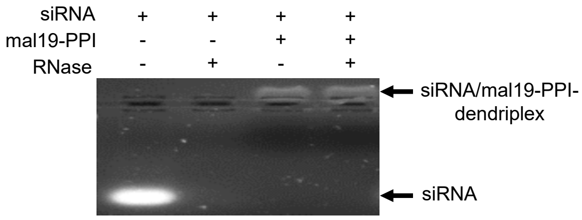

Figure S1. RNase protection assay on the resistance of siRNA/mal19-PPI-dendriplex (1:5) on RNase A/T1 Mix.

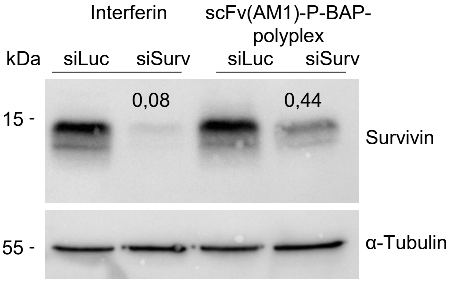

Figure S2. Targeted delivery of therapeutic siRNA using scFv(AM1)-P-BAP-guided polyplexes in PSCA-positive PC3 cells. Western Blot analysis showing knockdown efficiencies of Survivin in PC3^{PSCA} cells treated with scFv(AM1)-P-BAP-guided polyplexes containing siSurv or siLuc. As positive RNAi control, cells were transfected with siSurv or siLuc using the transfection reagent Interferin.

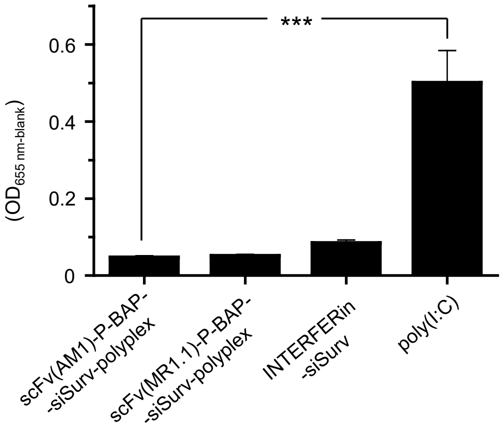

Figure S3. HEK-Blue$^{hTLR3/PSCA}$ cells were treated with scFv(AM1)-P-BAP-guided polyplexes containing siSurv. As positive control, cells were treated with poly(I:C). Induction of secreted alkaline phosphatase (SEAP) activity was assessed via optical density (OD) at 655 nm (n = 2, mean ± SD, ***$p < 0.001$).

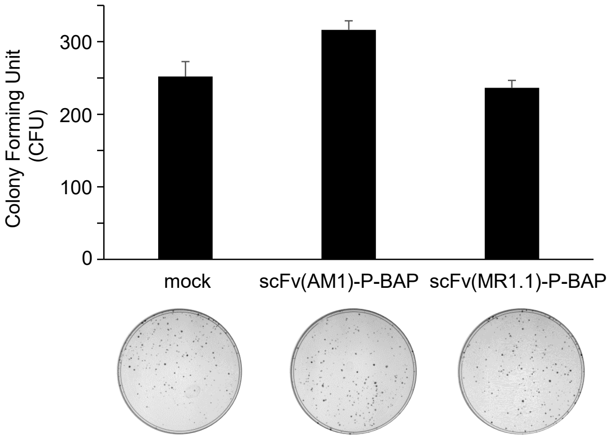

Figure S4. Clonogenic survival assay of PC3^{PSCA} cells after treatment with scFv(AM1)-P-BAP/neutravidin conjugates compared to scFv(MR1.1)-P-BAP/neutravidin conjugates (n = 3, mean ± SD).

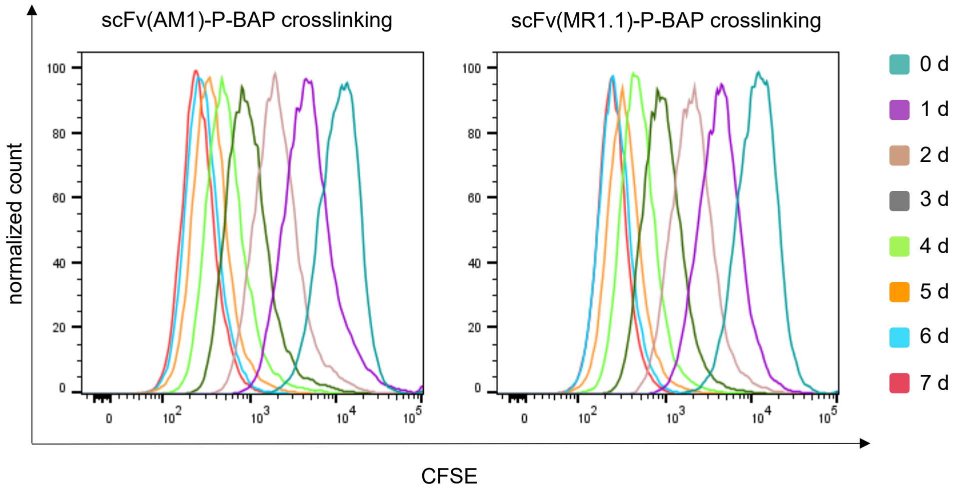

Figure S5. Carboxyfluorescein succinimidyl ester (CFSE)-based cell proliferation assay of PC3^{PSCA} cells after treatment with scFv(AM1)-P-BAP/neutravidin or scFv(MR1.1)-P-BAP-conjugates.
